# Single-Dose Lentiviral Mediated Gene Therapy Recovers CFTR Function in Cystic Fibrosis Knockout Rats

**DOI:** 10.3389/fphar.2021.682299

**Published:** 2021-05-18

**Authors:** Nicole Reyne, Patricia Cmielewski, Alexandra McCarron, Juliette Delhove, David Parsons, Martin Donnelley

**Affiliations:** ^1^ Adelaide Medical School, University of Adelaide, Adelaide, SA, Australia; ^2^ Respiratory and Sleep Medicine, Women’s and Children’s Hospital, North Adelaide, SA, Australia; ^3^ Robinson Research Institute, University of Adelaide, Adelaide, SA, Australia

**Keywords:** cystic fibrosis, lentival vector, nasal potential difference (NPD), Rat, gene therapy

## Abstract

Cystic fibrosis (CF) is a genetic disease caused by mutations in the CF transmembrane conductance regulator (*CFTR*) gene, resulting in defective ion transport in the airways. Addition of a functioning *CFTR* gene into affected airway cells has the potential to be an effective treatment for lung disease. The therapeutic efficacy of airway gene transfer can be quantified in animal models by assessing ion transport in the treated nasal epithelium using the nasal potential difference (PD) measurement technique. The nasal PD technique is routinely used in CF mice, however when applied to a recently developed CF rat model those animals did not tolerate the initial nasal PD assessment, therefore the procedure was firstly optimised in rats. This study evaluated the effect of lentiviral (LV)-mediated *CFTR* airway gene delivery on nasal PD in a CFTR knockout rat model. LV gene vector containing the *CFTR* gene tagged with a V5 epitope tag (LV-V5-*CFTR*) was delivered to the nasal epithelium of CF rats, and one week later nasal PD was analysed. This study demonstrated for the first time that LV-V5-*CFTR* treatment produced a mean correction of 46% towards wild-type chloride response in treated CF rats. Transduced cells were subsequently identifiable using V5 immunohistochemical staining. These findings in the nose validate the use of airway gene therapy for future lung based experiments.

## Introduction

Cystic fibrosis (CF) is a common, life-shortening, recessive genetic disorder, resulting from mutations in the cystic fibrosis transmembrane conductance regulator (*CFTR*) gene (Davis, 2006). CF affects multiple organs and despite symptomatic treatments and CFTR modulator therapies that improve quality of life it remains incurable, with lung disease the major cause of mortality (Carlon et al., 2017). The pathophysiology of CF results from a loss of CFTR chloride channel function and via its interaction with other ion channels such as the epithelial sodium channel (ENaC). Together these channels regulate chloride and sodium ion movement across the epithelial surface of many organs, maintaining osmolarity. In CF airways an imbalance of these ions creates altered movement of water, causing the mucus layer lining the airways to become dehydrated and vicious. The resulting viscous mucus is thick, difficult to clear, and the ideal environment for bacteria and other pathogens to grow. Thus, CF airways experience a continuous cycle of infection and inflammation, leading to permanent lung damage and ultimately respiratory failure (Chmiel et al., 2002).

Correcting CFTR dysfunction at the genetic level, by the addition of a functioning *CFTR* gene into affected airway cells, has the potential to be an effective treatment for CF lung disease. As CF is monogenic, addition of one correct copy of the *CFTR* gene into the cells should be sufficient to halt disease or prevent it if initiated early in life (Villate-Beitia et al., 2017). A range of viral and non-viral vectors can be used to facilitate the transfer of a gene to the airway epithelial cells. Lentiviral vectors (LV) are attractive vehicles as they can transduce dividing and non-dividing cells, integrate into the host genome providing long term expression, and generate little immune response (Castellani and Conese, 2010).

Our group has utilised a vesicular stomatitis virus type G (VSV-G) pseudotyped LV vector and two-step dosing procedure that involves conditioning the airways with lysophosphatidylcholine (LPC) prior to vector delivery. Using this approach, we have shown that nasal delivery of LV-*CFTR* can successfully correct CFTR function in a CF mouse model for up to 12 months after a single dose, and improves survival of the treated animals (Cmielewski et al., 2014). The nasal airway region is used because the ion transport defects in the CF mouse nasal airway epithelium closely resemble those of the human CF lung (Ferrari et al., 2002). Additionally, the nasal cavity is easily accessed, which makes it a suitable site to test ion transport function as a representation of the lower airways (Rowe et al., 2011). CF mouse models have predominantly been used in CF research, however they do not exhibit the human lung pathophysiology, so further lung investigations can be limited (McCarron et al., 2018; McCarron et al., 2020).

Nasal PD has been used as a CF diagnostic method in humans for over 25 years, as it is well tolerated (Knowles et al., 1981). It has been adapted for use in some CF animal models, particularly mice, to test the efficacy of various therapeutic strategies. A *CFTR* knockout (KO) rat model was recently developed by CRISPR/Cas9 gene editing as an alternative CF animal model for researchers (McCarron et al., 2020). Preliminary data from this model showed altered function of the nasal epithelial ion channels that is consistent with the CF profile (McCarron et al., 2020). Those preliminary nasal PD investigations were performed as non-recovery procedures due to rats displaying a heightened sensitivity to fluid in the airways and respiratory difficulties following the procedure, meaning that recovery was not possible. The initial nasal PD assessments in rats were performed in the same manner as for mice, with the animal hung by the dorsal incisors in a vertical position (Limberis et al., 2002). This orientation proved to be problematic, as all nasally delivered solutions collected in the lungs, resulting in respiratory distress. Other factors limiting the first nasal PD investigations in rats included the poor integrity of the existing nasal PD fluid delivery perfusion tubing arrangement. Therefore, the nasal PD measurement technique in rats needed to be optimised to achieve improved reliability so that the effect of treatments such as airway gene-addition therapy could be assessed.

In this study we firstly optimised the technique of performing nasal PD measurements in rats to test ion channel function. We then investigated whether a single dose of our LV-V5-*CFTR* vector delivered after conditioning with LPC recovers CFTR function in the nasal epithelium of CF KO rats.

## Methods

### Animals

This project was conducted under the approval from the University of Adelaide Animal Ethics Committee (M-2017-056). All rats were maintained in conventional cages with a 12-h light/dark cycle. Food and water were provided *ad libitum*, with all rats receiving a 50:50 mix of normal and high fat (10%) rodent chow. CF rats received water containing 4.5% ColonLytley (Dendy Pharmaceuticals, Australia) to minimise CF related gut obstructions. Female and male CF KO rats (510X genotype) (McCarron et al., 2020) were used.

### Nasal PD

Ion channel function was measured using a newly optimised nasal PD technique adapted from mouse protocols (Limberis et al., 2002; Cmielewski et al., 2010) and early CF rat characterisation studies (McCarron et al., 2020). Rats were anesthetized with 60–75 mg/kg of a mixture of ketamine (Ceva, Australia) and medetomidine 0.4 mg/kg (Ilium, Australia), delivered by intraperitoneal injection. Rats were then placed on a customised nasal PD platform, in the prone orientation. Upper incisors were hooked over a wire loop, resulting in their mouth being open, rather than vertical with an endotracheal tube as in the initial rat PD method.

Our old PD system was constructed using long thin polyethylene tubes and cut-down needles sealed with superglue or silicone sealant where connections were required. This perfusion tubing had poor integrity, with bubbles and blockages regularly interrupting the continuous electrical circuit, leading to erroneous PD readings. New perfusion lines (Instech, United States) were made using 25 ga tubing (#BTPE-25), PinPorts (#PNP3F25) and PinPort connectors (#PNP3M-50) designed for minimal dead space volumes, as well as three-way tubing connectors (#SCY25), to create an enclosed system that prevented bubble formation. A micropositioner provided fine tip positioning and firmly held the nasal cannula in place, thus limiting movement while the cannula sat in the nose. Excess perfusion fluid coming out of the nasal cavity and/or mouth was absorbed with tissue. A 15 ml centrifuge tube was placed under the neck to create a slight downwards bend of the head and nose tip to help prevent fluid from moving distally and entering the trachea. The cannula was inserted 4–7 mm into the nasal cavity, based on identification of the location of nasal respiratory epithelium using dissected rat heads (not shown) and comparison to rat nasal diagrams (Mery et al., 1994). The reference electrode inserted subcutaneously into the abdomen, and connected to calomel electrodes (Hg2Cl2 in 3 M KCl, Cole-Parmer Instruments, United States) (Limberis et al., 2002; Cmielewski et al., 2010).

The optimised nasal PD method was then tested in wild-type (WT) and CF KO rats. PD data was recorded using a optically isolated high-impedance millivolt meter (ISOMIL, World Precision Instruments) connected to an eight channel Powerlab, combined with Labchart software (Version 8, ADInstruments).

The nasal cavity was perfused at a rate of 10 μL/min with the following solutions: 1) normal Krebs-Ringer buffer (basal KRB), 2) KRB containing 100 µm amiloride, 3) low chloride KRB with 100 µm amiloride, and 4) low chloride KRB containing 100 µm of amiloride and 100 µm of isoproterenol. Each solution was perfused for 10–15 min until a plateau of 1–2 min was obtained. The change in PD (ΔPD) was calculated by subtracting the PD values at the plateau for each different solution. PD traces were rejected if they were non-responders.

### Vector Production

This study employed a third generation, HIV-1 derived, VSV-G pseudotyped, *CFTR* gene vector with a V5 epitope tag located on the N-terminus. Gene expression was driven by the EF1α promoter (CCL-EF1α-V5-CFTR) The vector will be referred to as LV-V5-*CFTR*. LV vector was produced as described previously (Rout-Pitt et al., 2018). Briefly, HEK 293T cells seeded in 10-layer cell factories were transiently transfected using a five-plasmid system and calcium phosphate co-precipitation. Two days post-transfection the vector supernatant was harvested and purified by anion exchange chromatography, followed by ultracentrifugation. Vector was formulated in 0.1% heat-inactivated rat serum in 0.9% saline.

### Vector Titre

The titre of the LV-V5-*CFTR* vector was determined by flow cytometric detection of V5 expressing-cells. HEK 293 T cells were transduced with a serially diluted (1:2,500–1:320,000) vector followed by collection 72 h post-transduction. Cells were fixed in 4% paraformaldehyde, permeabilized with ice cold 90% methanol, and resuspended to a concentration of 1 × 10^6^ cell/mL for staining. Primary antibody rabbit anti-V5 Tag (#13202, Cell Signaling Technologies, United States) and secondary antibody goat anti-rabbit IgG (Alexa Fluor 568) (a11011, Invitrogen, United States of America) were added to the cells separately for 1 h and 30 min respectively at room temperature. Cells were washed between antibody staining and after, then resuspended in PBS. Cells were analysed using flow cytometry (LSRFortessa^TM^; Becton Dickinson, United States). The proportion of V5-positive cells was used to calculate functional titre in transducing units per ml (TU/mL). A titre of 8 × 10^6^ TU/ml was used for rat nasal dosing.

### Nasal Vector Dosing

Rats were anaesthetised as described above. Lubricating eye ointment was placed onto the eyes to minimise drying. Rats were placed on a heating mat for the duration of the procedure and recovery period. Instillations were performed into the right nostril using a micropipette and thin gel tip (No: 5,242,956.003, Microloader, Eppendorf, Germany). Airway conditioning was performed with 2 × 5 µL aliquots of 0.3% LPC (#L4129 Sigma, United States), delivered 1 h prior to 2 × 10 µl aliquots of LV-V5-*CFTR* vector. Anaesthesia was reversed using 1 mg/kg atipamezole (Jurox, Australia). Baseline PD was performed at least one week before LV-*CFTR* delivery on the untreated nostril. One week after LV-*CFTR* delivery, PD measurement was performed on the treated nostril. All PD tracings were interpreted by an experienced assessor blinded to the treatment.

### Immunohistochemistry

Rats were humanely killed by intracardiac sodium pentobarbital overdose (300 mg/kg) while under anaesthetic. To examine the nasal passageways the heads were collected and immersion fixed in 10% neutral buffered formalin followed by decalcification in 25% EDTA (pH 7.4) for 7–14 days at 37°C. Heads were cut, paraffin-embedded, sectioned at 5–7 μm, and deparaffinised using standard histological procedures. Antigen retrieval was performed using 10 mM sodium citrate buffer (pH 6.0) for 20 min followed by permeabilisation in 0.3% Triton-X 100 in PBS for 10 min. Sections were blocked for 1 h at room temperature with 1% BSA in PBS and then incubated with goat anti-V5 primary antibody (1:300) (ab95038, Abcam) in 1% BSA + 0.05% Tween overnight at 4°C. Sections were rinsed and incubated in donkey anti-goat (IgG) Alexa Fluor 568 (1:400) (ab175704, Abcam) made in 1% BSA and 0.1% Tween incubated at room temperature for 1 h. Samples were washed with 0.1% Tween-PBS, counterstained with DAPI for approximately 10 min, washed, and mounted with ProLong^TM^ Diamond antifade mounting media (#P36961, Life Technologies, United States). Slides were visualised for the presence of V5 staining using an Olympus BX51 Research grade optical microscope DIC with AnalySIS Life Sciences software.

### Statistical Analysis

Statistical analysis was performed using Graphpad Prism V9.0 (GraphPad Software Inc.). Statistical significance was set at *p* ≤ 0.05 and a statistical power greater than 0.80 was required. Paired t-tests were used to compare the treated and untreated nostril in each rat. In text values are reported as mean ±SD.

## Results

### Nasal PD Assessment Was Optimized in Rats

To improve nasal PD assessments in rats we developed an optimised testing method. In the preliminary nasal PD studies (McCarron et al., 2020), rats readily inhaled the nasally delivered solutions into the lung, often leading to respiratory distress. This was a critical limitation, as rats could not be recovered from anaesthesia following the procedure. Several measures aimed at reducing that original procedure-associated complication were successfully trialed, including placing the rats in their naturally prone position, and absorbing the excess fluid from the nasal cavity. Using tubing with a smaller bore diameter reduced the volume of fluid in the system and enabled perfusion fluids to be rapidly changed, without excessive fluid delivery to the nose. Subsequently, rats did not show signs of respiratory distress and no mortality was associated with the nasal PD procedures.

### Confirmation of WT and CF Rat Nasal PD Profiles

**FIGURE 1:**
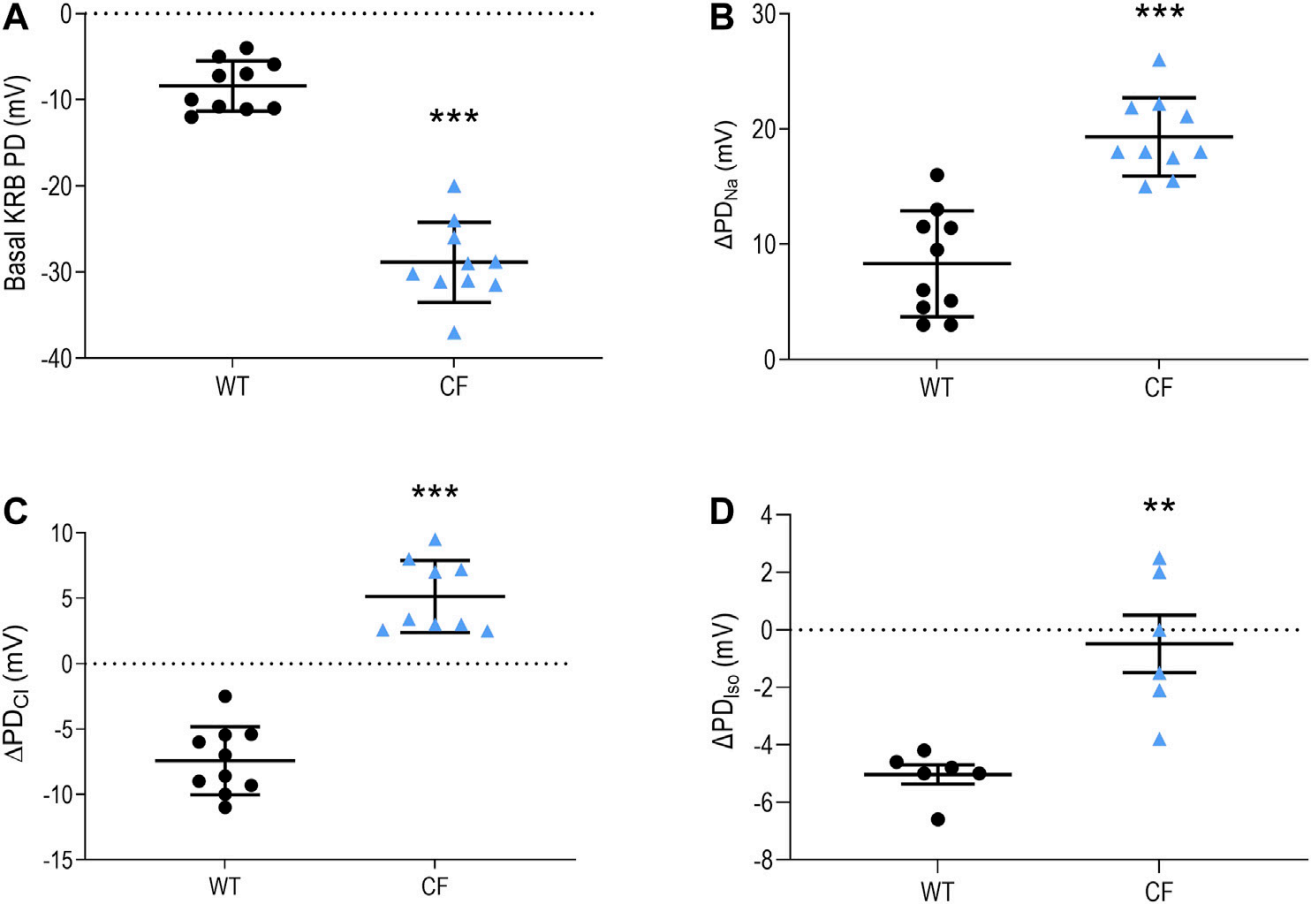
Nasal PD measurements from WT and CF KO rats. Nasal PD measurements in WT and CF KO rats for **(A)** basal KRB **(B)** the ΔPD_Na_ **(C)** ΔPD_Cl_ and **(D)** ΔPD_Iso_ (*n* = 6–10 animals/group; **p ≤ 0.01, ***p ≤ 0.001, unpaired *t*-test). Data represented as the mean −/+ SEM.

This study provides an improved characterisation of the airway bioelectric profile in our CF KO rats (McCarron et al., 2020), with the greater power revealing that there was a statistically significant difference in the isoproterenol response between WT and KO CF rats (*p* ≤ 0.01, Figure 1). Under basal conditions, CF KO rats demonstrated a significant negative PD when compared to the WT rats. When the ENaC inhibitor amiloride was perfused, CF KO rats exhibited a more enhanced depolarisation response than WT rats, indicating increased luminal retention of sodium. Low chloride and isoproterenol solutions produced either no response or a small depolarisation in CF (whereas a hyperpolarisation was observed in WT rats) suggesting the absence of CFTR-mediated chloride transport.

### Ion channel function can be restored in nasal epithelium of CF KO rats after treatment with LV-V5-CFTR

**FIGURE 2:**
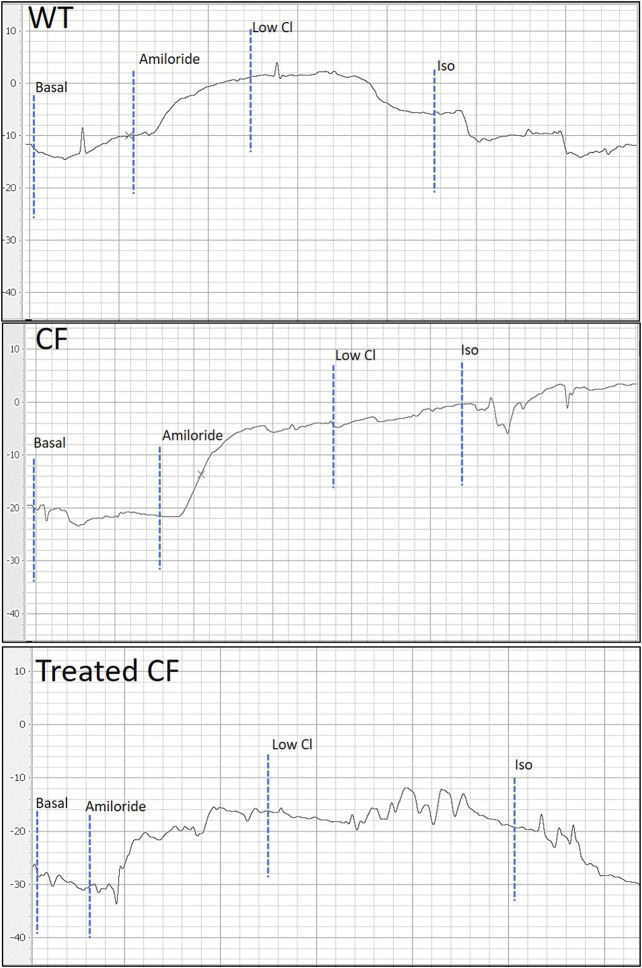
Representative nasal PD tracing of WT, CF KO and treated CF KO rat. When compared to WT, CF KO rats demonstrate classic CF electrophysiological defects in nasal respiratory epithelium. Treated CF KO rats demonstrate a PD trace showing the corrected bioelectrical defect.

Figure 2 depicts representative PD traces from WT, CF KO and a treated CF KO rat, showing the correction of the bioelectrical in the treated CF KO rat.

**FIGURE 3:**
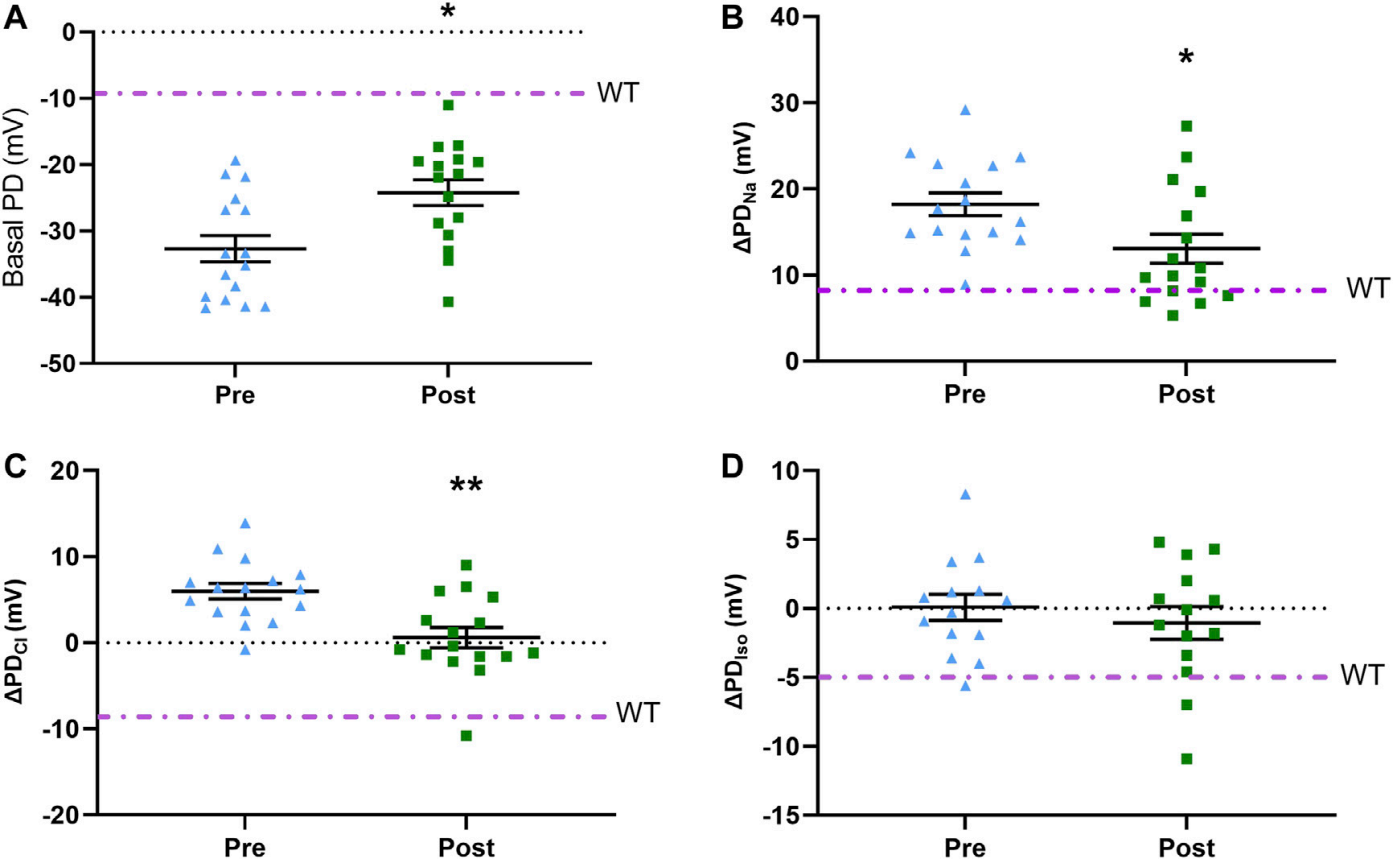
Pre-treatment and post-treatment nasal PD results following LV-V5-*CFTR* vector delivery to nasal epithelium of CF KO rats. Nasal PD measurements in CF KO rats for **(A)** basal KRB **(B)** ΔPD_Na_ **(C)** ΔPD_Cl_, and **(D)** ΔPD_Iso_. Dot/dash line indicates the WT average (*p ≤ 0.05, **p ≤ 0.01, paired *t*-test; *n* = 14–16). Data represented as the mean with −/+ SEM.

Seven days after delivery of LV-V5-*CFTR* to CF KO rats (*n* = 16), the basal KRB (−24.2 ± 7.8 mV) response exhibited a significantly smaller depolarisation compared to pre-treatment (−32.8 ± 7.9 mV), which was closer to a WT response (*p* ≤ 0.05) (Figure 3). The amiloride response in treated (13.0 ± 6.7 mV) CF KO rats had an improvement of 51% toward the WT value when compared to the pre-treatment (18.2 ± 5.3 mV) (*p* ≤ 0.05). Treated CF KO rats also showed a more negative low chloride response (0.6 ± 4.7 mV), which was significantly different from the pre-treatment response (5.9 ± 3.6 mV) (*p* ≤ 0.01) (Figure 3). The isoproterenol response did not reach statistical significance between pre- and post-LV treatment (0.08 ± 3.6 and −1.5 ± 4.4 mV respectively).

**FIGURE 4:**
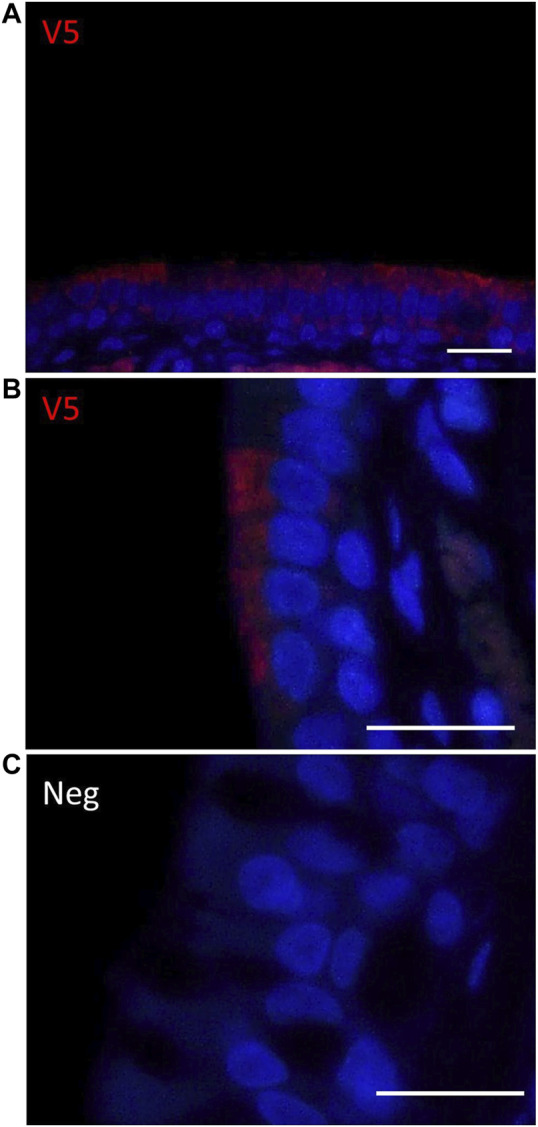
Immunohistochemical detection of V5-*CFTR* in the rat nasal epithelium. **(A,B)** V5 IHC enables localisation of *CFTR* (red) in the right nostril (treated) one week after delivery of LV-V5-*CFTR.* **(C)** No staining was observed on the left (untreated) side of the rat nose. Scale bar 20 μm.

Immunostaining with an anti-V5 antibody demonstrated the presence of the transgene in the right side (treated) of the nasal epithelium one week post-delivery, whereas no staining was observed in the left nostril (untreated). V5 positive staining was detected in the ciliated cells of the nasal epithelium (Figure 4).

## Discussion

Nasal PD measurement methods have been developed for use in mice, ferrets and pigs, and enable reliable repeated measurements in the same animal with low procedure-associated mortality (Beka and Leal, 2018; Kaza et al., 2017; Salinas et al., 2004). We have demonstrated that our newly optimised technique and equipment for conducting electrophysical measurements, can provide a reliable tool for evaluating airway gene therapy effects in CF rat nasal airways. The nasal PD baseline data collected from WT and CF KO rats confirmed that the CF KO rat displays a bioelectrical defect consistent with the CF profile (McCarron et al., 2020).

The nasal PD response to the low chloride gradient is used to both discriminate non-CF from CF animals (Figure 2), and more commonly to assess the response to therapeutics *in vivo* (Leonard et al., 2012). It has been suggested that a relatively small number of gene corrected airway cells (10%) can restore transepithelial chloride function to therapeutically significant levels, potentially providing dramatic improvements in pulmonary function (Johnson et al., 1992; Marquez Loza et al., 2019). Moreover, those CF patients with ‘mild’ mutations and residual CFTR function have less severe lung disease (Griesenbach et al., 2008) indicating that not all cells are required to be corrected. Our previous CF mouse study showed a 12–54% correction in PD towards WT CFTR function as assessed by chloride transport (Cmielewski et al., 2014), and in this present rat study, a mean of 46% correction was observed. The isoproterenol response did not reach statistical significance in this study, however larger studies or a higher level of correction may provide sufficient power to detect a difference. Importantly, this study showed for the first time that a single delivery of LV-V5-*CFTR* could achieve *in vivo* correction of nasal airway epithelium ion transport in a CF KO rat model.

CF epithelial ion transport is characterised by a greater hyperpolarisation (more negative) measurement of basal KRB due to the increased activity of ENaC, a large depolarisation in the presence of a sodium channel blocker, and a small or absent response to low chloride and isoproterenol solutions. Gene therapy efforts have focused on the correction of chloride function and some suggest that the direct interaction between CFTR and ENaC should be normalised for therapeutic outcomes (Johnson et al., 1995). Johnson et al. indicated with *in vitro* studies using ALI cultures that a majority of the cell monolayer is required to be expressing *CFTR* for ENaC transport to be corrected (Johnson et al., 1995), while another *in vitro* study suggested that 60% of ciliated cells may be enough to normalise sodium ion transport (Zhang et al., 2009). We noted there was a statistically significant mean correction of 51% towards WT in the amiloride response after LV vector treatment in CF rats. This may suggest that the sodium hyperabsorption defect present in CF rats may have been improved, however, further studies utilising Ussing chamber assessments are needed to link altered sodium absorption to the insertion of a functioning CFTR channel.

The HIV-1 virus-based vector system has been extensively modified to improve safety and efficacy for use in gene therapy (Milone and O’Doherty, 2018). While our previous *CFTR* airway gene-addition studies employed second generation LV vectors with internal viral promoters (e.g., SV40), for the first time this study used a third generation vector with an EF1α promoter, tagged with V5 for immunodetection. For these reasons this vector system is more appropriate for clinical application than those used in our previous mouse studies (Cmielewski et al., 2014). Importantly, the use of the V5 tag did not interfere with functionality of the CFTR protein, as demonstrated by significant correction of bioelectric defect as measured by nasal PD.

This study had some limitations. Firstly, it was designed to be a short-term assessment and did not investigate the ability to achieve long term correction of nasal ion transport. For gene therapy to be successful the expression of *CFTR* needs to be permanent and life long, and this study sets the stage for future long-term investigations in CF rats. Assessing persistence of gene expression over long durations in CF rats and the ability to re-dose if correction wanes are all next steps. Secondly, the total number of *CFTR* transduced cells was not quantified here, but in future studies where histological outcomes are not required the nasal epithelium could be removed, the cells dissociated and the V5 positive cell population quantified by flow cytometry. CFTR protein expression could also be further examined by Western blot. Thirdly, the use of a non-CFTR LV vector control could be employed to verify that the delivered *CFTR* gene was responsible for the effects observed here. Lastly, a CFTR inhibitor might be used to further discriminate CFTR function, although one study suggests that CFTRinh-172 does not have an effect in rats (Dreano et al., 2019).

In summary, using our optimised PD protocols we showed successful correction of the bioelectrical defect in our newly established CF KO rat model for the first time. Future studies will focus on assessing long-term CFTR correction, to help validate the effectiveness of this LV vector for translation to the clinic as a potential and fundamental treatment for CF lung disease.

## Data Availability

The raw data supporting the conclusions of this article will be made available by the authors, without undue reservation, to any qualified researcher.
